# Effectiveness of Virtual Reality Therapy on Balance and Gait in the Elderly: A Systematic Review

**DOI:** 10.3390/healthcare12020158

**Published:** 2024-01-09

**Authors:** Daniel Rodríguez-Almagro, Alexander Achalandabaso-Ochoa, Alfonso Javier Ibáñez-Vera, Jorge Góngora-Rodríguez, Manuel Rodríguez-Huguet

**Affiliations:** 1 Department of Nursing, Physical Therapy and Medicine, University of Almería, 04120 Almería, Spain; dra243@ual.es; 2 Department of Health Sciences, Faculty of Health Sciences, University of Jaén, 23071 Jaén, Spain; aaochoa@ujaen.es (A.A.-O.); ajibanez@ujaen.es (A.J.I.-V.); 3 Department of Nursing and Physiotherapy, University of Cádiz, 11009 Cádiz, Spain; manuel.rodriguez@uca.es

**Keywords:** virtual reality, postural balance, conventional balance training, exercise

## Abstract

Virtual reality (VR) therapies are presently utilized to treat physical and cognitive impairments among elderly people. This systematic review aims to collect the most recent evidence on the effectiveness of VR in improving balance and gait among healthy elderly individuals, in comparison with other therapies. A literature search was conducted using the PubMed, SCOPUS, PEDro, and WoS databases, by selecting randomized clinical trials that evaluated balance, both static and dynamic, as well as gait in a population of healthy older adults who underwent virtual reality therapy. The methodological quality of the studies was assessed using the PEDro scale. After eligibility criteria were applied and duplicates were removed, 20 studies were selected out of 1705 initially identified. The present systematic review concludes that virtual reality therapy is more effective than minimal intervention or usual care in enhancing static balance, dynamic balance, and gait in healthy elderly individuals. Moreover, virtual reality therapy yields better outcomes compared to traditional balance training and physical exercise in improving balance and gait in this demographic. However, both methods have shown effectiveness.

## 1. Introduction

Due to the effects of aging, the body and mind undergo degenerative changes that can negatively impact daily activities. This can result in mobility issues, reduced independence, and a decline in psychological well-being. Ultimately, this leads to an increase in healthcare costs [1].

Balance and gait are crucial factors for the well-being of elderly individuals. Around 13% of adults aged 65–69 experience issues with balance, a number that increases to 46% for those over 85 years old [2]. Similarly, it is estimated that 35% of non-institutionalized adults over 70 years of age have gait disorders [3], which increases their risk of institutionalization and death by 2.2 times compared to those without these disorders [3]. Additionally, balance and gait disturbances are associated with a higher risk of falls [4]. About one third of non-institutionalized adults over 65 years old experience a fall during the year [5,6,7]. Injuries from falls, particularly hip fractures, significantly contribute to the mortality burden of elderly individuals and represent the leading cause of injury-related accidental death among those aged 65 or older. Consequently, falls among older adults have emerged as a critical public health issue [8,9] and an international health priority [10].

For the past few decades, fall prevention programs have gained attention in the field of prevention and public health. These programs range from multidisciplinary approaches involving physicians, pharmacists, physiotherapists, nurses, and social workers to structured exercise programs [11]. Studies have demonstrated that regular physical activity in older adults can improve muscle strength and balance, leading to enhanced performance in daily living tasks and decreased risks of falls, fractures, chronic illnesses, and related mortality [6,12]. Available evidence suggests that exercise programs comprising physical training [13,14], strengthening exercises [15], aerobic exercise [16], Tai Chi [17], and balance training [18] are the most efficient approaches to improving muscle strength, endurance, gait, and balance in older adults. These programs have been recognized as the optimal strategy to prevent and decrease the likelihood of falls [6,11]. However, these programs have a downside: low adherence [19]. Studies suggest that insufficient exercise intensity or poor adherence to the program can reduce its effectiveness [20,21].

The link between a higher level of treatment adherence and improved functional outcomes for balance and fall prevention has been confirmed [22]. Interactive video games have shown potential as an effective therapy for fall prevention [23], used for balance evaluation and rehabilitation [24]. Their use has been proven to increase adherence by over 30% compared to conventional exercise therapies [25].

Virtual reality therapy (VRT) offers numerous benefits. VRT positively affects participants’ motivation and enjoyment [26,27], ultimately leading to increased engagement with the therapy [28]. Patients focus on the game during treatment, forgetting physical deficits and creating a pleasant experience. This factor could lead to long-term functional improvement because the increase in attendance, and thus the increase in the completion of a sufficient number of sessions, would be crucial for inducing neural plasticity processes and facilitating motor learning [28]. Furthermore, VR offers the ability to create and maintain an environment in which the presence of the patient can be physically projected and with which the patient can interact [29]. In this way, VR allows for the simulation of real-world environments in which one can safely interact in real time, providing an environment for patients to practice therapeutic tasks that would otherwise not be feasible in the real world due to resource limitations or safety concerns. Another aspect is the ability of VR to provide visual, auditory, and/or haptic feedback that allows the patient to make adjustments in response to positive or negative feedback during task performance, thus facilitating motor skill learning [29,30,31]. In addition, linking positive feedback to successful therapeutic task performance may provide motivation and encouragement for individuals to engage in rehabilitation therapy, thereby increasing patient adherence [28,29,31].

Virtual reality (VR) therapies are currently being used to address physical and cognitive dysfunctions in elderly populations [32]. Despite variations in training protocols, such as the exercise type, repetition count, session length, and intervention duration, the results demonstrate potential advantages. Positive effects on balance parameters have been observed in older adults, including improvements in both static and dynamic balance, as well as a reduction in the fear of falling and an increase in lower limb strength. This systematic review aims to gather the most recent evidence examining the effectiveness of VR compared to other therapies in enhancing the balance and gait of healthy elderly individuals.

## 2. Materials and Methods

The systematic review was conducted in accordance with the guidelines outlined in the PRISMA (Preferred Reporting Items for Systematic Reviews and Meta-Analysis) Statement by Page et al. (2021) [33]. Additionally, the methodological recommendations put forth by Higgins et al. (2011) [34] in the “Cochrane Handbook for Systematic Reviews of Interventions” were considered in a complementary manner. In addition, we registered the protocol for this review with the registration number CRD42023387025 in PROSPERO, the International Prospective Register of Systematic Reviews.

### 2.1. Search Strategy and Data Sources

The literature search for this systematic review was carried out between December 2022 and May 2023 by two authors (M.R.-H. and J.M.G.-R.) in the following databases: PubMed, SCOPUS, PEDro (Physiotherapy Evidence Database), and Web of Science (WoS). Additionally, the authors conducted a search in the reference lists of the full-text articles obtained through the original search strategy, as well as in the gray literature and expert documents. The research question was formulated using the PICOS system proposed by the Cochrane Library [34] to establish the search strategy: (1) population, healthy older adults without known pathology; (2) intervention, VR-based therapy; (3) comparison, balance therapy, exercise or no intervention; (4) outcomes, balance, static and dynamic, and gait; and (5) study design, randomized clinical trial (RCT). The search strategy included medical subject headings (MeSH terms), the EBSCOhost thesaurus, and standardized terms established as descriptors in health sciences (DeCS), as well as free terms such as “ancient”, “exergame”, and “balance disorders”. To conduct an ideal search process, we joined the chosen terms using Boolean operators AND and OR, following the search criteria of each database. In addition, we did not establish any filters concerning language, the availability of full-text resources, or access to them in the current search. Any disagreements regarding the search were resolved through consultation with a third party possessing expert in bibliographical searches, D.R.-A. Duplicate studies were excluded during the next step of study selection. The search strategy utilized for each database is provided in Table 1.

### 2.2. Selection of Studies and Eligibility Criteria

For the identification of potentially eligible studies, 2 blinded reviewers (M.R.-H. and J.M.G.-R.) independently screened the titles and summaries of all references identified by the search strategy. Disagreements that arose during the selection process were resolved by a third reviewer (A.J.I.-V.).

Inclusion criteria were defined as: (1) those studies that were randomized clinical trials (RCT); (2) composed of healthy older adults; (3) in which the experimental group had been approached with VR therapy; (4) to assess balance and gait after the intervention.

On the other hand, we excluded: (1) articles the design of which was different from RCTs, including pilot studies; (2) those in which the mean age of the sample was less than 65 years; or presented any type of pathology; and (3) those that obtained a score lower than 6 on the PEDro scale, with the aim of drawing relevant conclusions.

### 2.3. Data Extraction

Two independent reviewers (M.R.-H. and J.M.G.-R.) gathered information from the studies that were included. Discrepancies were resolved by the participation of a third author (A.J.I.-V.). For each study chosen for this review, we collected the author’s name and publication year, study type, study population, details of both the intervention and control groups, intervention procedure including the type of intervention in both groups, frequency and duration of sessions, number of evaluations, main variables, measurement instruments, and results of the intervention in both groups.

### 2.4. Variables

Balance, including both static and dynamic components, was the primary focus of this study. The methods employed to measure balance involved a range of tests, such as the MiniBEST test, the Berg Balance Scale (BBS), the Tinetti-POMA scale, the one-leg standing test (OLS), the Functional Reach Test (FRT), the Timed Up and Go test (TUG), and the posturography platform, objectively assessing balance. Less frequently evaluated secondary variables in the studies involved gait, stability, fear of falling, cognitive function, and upper and lower extremity strength.

### 2.5. Quality Assessment

The methodological and evidence quality of every outcome in each study was autonomously evaluated by two reviewers, A.A.-O. and A.J.I.-V.

To evaluate the methodological quality and risk of bias of the included studies, we employed the PEDro Scale. This tool comprises 11 items (I) that can be answered as “yes” if the item is met, or “no” if not. By adding the scores of items 2 through 11 (item 1 is excluded as it pertains to external validity [35,36]), a total score between 0 (high risk of bias) and 10 (no risk of bias) can be obtained [37]. The methodological quality can be rated on a scale from excellent (10–9 points) to good (8–6 points), moderate (5–4 points), and low (3–0 points). Regarding the risk of bias, items 2 and 3 refer to selection bias, items 5 and 6 refer to performance bias, and item 7 is related to detection bias [37].

## 3. Results

### 3.1. Study Slection

**Figure 1:**
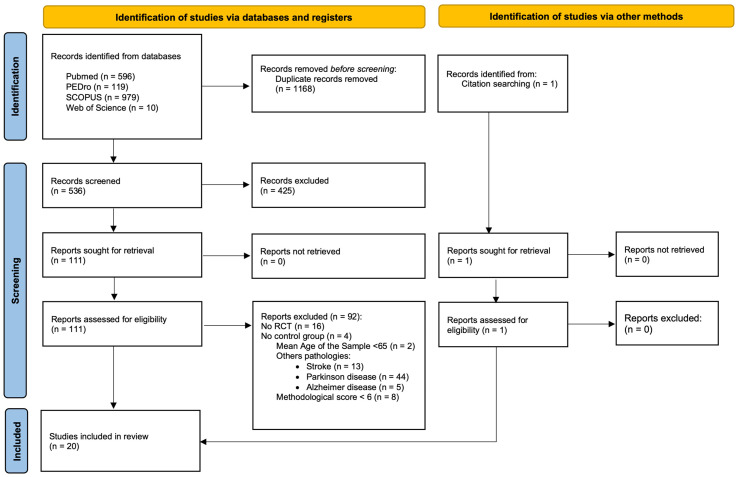
PRISMA flow diagram for the study selection process.

A total of 1705 publications were identified through the bibliographic resources explored in the initial search. After eliminating duplicate studies and applying the eligibility criteria, 20 articles were selected for this systematic review. Figure 1 displays a detailed overview of the selection process.

### 3.2. Methodological Quality Assessment

The PEDro score for each study included in the review is shown in Table 2. The included studies showed a good methodological quality and moderate risk of bias (PEDro score 6.9 ± 0.9 points). A total of nine studies scored 6 out of 10 [38,39,40,41,42,43,44,45,46], four studies scored 7 out of 10 [47,48,49,50], and seven studies scored 8 out of 10 [51,52,53,54,55,56,57]. In general, the studies in this review have a very low risk of selection bias, as only two articles do not meet item 3 [45,50]. In contrast, only three articles meet items 5 and 6 [51,54,56], and one meets item 6 but not item 5 [52]; the rest do not meet either of these two items, so the risk of over- or underestimating the results of the studies in this review is very high. Finally, only four studies do not meet item 7 [38,39,43,46], so the risk of detection bias is very low.

### 3.3. Characteristics of the Studies Included in the Review

A total of 939 subjects were analyzed across 20 included studies. Among them, 468 received VR-based therapy in the experimental group (EG), and 471 were in the control group (CG). The mean age of patients in the EG was 78.64 years, the mean age of patients in the CG was 75.86 years, and the mean age for all participants was 77.25 years. The intervention typically lasted for a period of 4 to 8 weeks in most studies, except for one study that had a duration of 16 weeks [38]. There was an average of two to three sessions per week, with the exception of the study conducted by Karahan et al. [41], which had five sessions per week. The average session duration for both the experimental group (EG) and the control group (CG) ranged from 30 to 60 min, except for one session that lasted about 6 min. Table 3 highlights the key characteristics of the studies encompassed in this review.

### 3.4. Effect of the Intervention

The reviewed studies showed great variability in terms of intervention protocols, the duration of sessions and interventions, and the VR devices and software used. Similarly, a wide variety of balance assessment methods were evaluated. For these reasons, and given the great heterogeneity in terms of measurement procedures and instruments, and with the aim of facilitating the understanding of the results obtained, the articles were grouped according to the intervention carried out in both groups.

#### 3.4.1. Virtual Reality Therapy Compared to Usual Care or Minimal Intervention

A total of 9 [38,39,40,42,44,51,52,53,55] of the 20 studies examined in this review assessed the impact of virtual reality (VR) program-based balance therapy compared to usual or minimal care interventions. The terms “usual care” or “minimal care interventions” refer to activities that are not initially assumed to have a significant impact on balance or gait, such as memory, mobility or occupational programs, nursing care, usual physical activity, or no intervention at all. The primary objective was to measure the effectiveness of the training program. The main characteristics and results of the studies included in this section are presented in Table 4.

Adcock, M. et al. [38] conducted an RCT where they set as their main objective to evaluate the effects of multicomponent exergame-based home training on physical and cognitive functions in healthy older adults. The study included thirty-one older adults, with an average age of 73.9 years, divided into two groups. The experimental group (EG) underwent 4 months of strength, balance, and cognitive training utilizing the Active@Home exergames program. The training consisted of three 30–40-min sessions per week, while the control group (CG) continued with their usual daily activities. After the intervention, improvements were noted in certain parameters related to executive function. The results are presented in Table 4.

Benítez-Lugo, M.L. et al. [51] suggest a regimen of 16 intervention sessions, consisting of 30-min sessions twice per week. The Wii Fit exercise program, using the Nintendo Wii and the Wii Balance Board platform, incorporated two games designed to enhance aerobic and balance capabilities in treating the experimental group of a trial comprising 46 individuals with a mean age of 72.65 years. The results revealed significant differences between the groups across the majority of evaluated variables. The findings from the intergroup analysis indicated that the experimental group exhibited statistically significant differences compared to the control group in terms of balance, gait, fall risk, attention, and cognitive status. The results are shown in Table 4.

Campo-Prieto, P. et al. [39] examined a group consisting of 24 older adults with a mean age of 84.96 years. The group was divided into a control group that received no additional treatment and an experimental group that undertook 30 6-min sessions (three sessions per week for ten weeks) of exercise-based boxing in a virtual gym space while wearing immersive virtual reality goggles. The results of the intergroup analysis indicated that the experimental group showed significant differences in balance, gait, and lower limb function compared to the control group, both post treatment and at follow-up. The results are reported in Table 4.

Delbroeck, T. et al. [40] questioned the efficacy of dual-task-based VR training in improving cognition, balance, and dual-task performance in older adults. They conducted an RCT on 20 older adults with a mean age of 87.2 years. The study group received 12 sessions of cognitive motor training with a VR device called BioRescue, while the control group continued with their usual physical therapy care. While the study did not examine variances between the interventions given to each group, the outcomes demonstrated notable advancements for the EG in three gait domains. The results are displayed in Table 4.

Gallardo-Meza, C. et al. [52] compared the effects of a 4-week exercise program on an experimental group of 37 women with a mean age of 68.1 ± 3.3 years to a control group of 35 women with a mean age of 69.2 ± 3.7 years. The exercise program consisted of two 30-min weekly sessions with the Wii Fit Plus program, using the Wii Balance Board and Wii Nunchuk devices (Nintendo Wii, Tokyo, Japan), preceded by a 5-min warm-up. The study demonstrated statistically significant relationships between the static balance of both legs, timed standing and walking, and average speed in the five-repetition sit-to-stand test. The EG showed significant improvements in muscle fitness, right and left leg static balance, the timed stand-and-walk test, and the five-repetition sit-to-stand test. The results are presented in Table 4.

Gomes, G.C.V. et al. [53] conducted a randomized controlled trial on 30 older adults with a mean age of 84 years to examine the feasibility, safety, and acceptability of playing interactive video games using Nintendo Wii Fit Plus™ (NWFP), as well as its impact on balance, gait, cognitive function, mood, and the fear of falling. The EG underwent 14 individual exercise training sessions with Northwestern Fitness and Performance (NWFP) that were customized to meet the patient’s unique requirements. All sessions were supervised by a licensed physiotherapist. Conversely, the CG was provided with a booklet that solely presented the World Health Organization’s recommendations on physical activity. The findings indicated a significant improvement in favor of the EG regarding static balance. This study found that there was a significant improvement in balance in EG immediately at post treatment (*p* = 0.003), but it was not maintained over time, as no significant differences were noted at follow-up (*p* = 0.053). The study also observed improvements in gait (FGA). These improvements were observed immediately after the intervention (*p* = 0.006) and maintained over time (*p* = 0.007). The results are shown in Table 4.

The study conducted by Kim, S.H. et al. [55] involved two experimental groups; one received virtual reality intervention, the other received motor imagery intervention, and a CG received no added treatment. The total sample comprised 34 older adults with a mean age of 79.68 years. The virtual reality exercise program comprised 30-min sessions held three times a week for 6 weeks, incorporating balance and rhythm games on the Nintendo Wii. Between-group analysis revealed that the EG exhibited significant differences in open-eye balance sensation compared to the CG after treatment. The results are described in Table 4.

Another study in this field was that of Lee, Y. et al. [42], which aimed to analyze how 3D technology in virtual reality training could enhance balance and strengthen lower extremities among older adults. The study involved 40 older adults, with an average age of 76.5 years, who were randomly split into two groups. Both groups underwent three sessions of fall prevention education, while the EG group received an additional 12 60-min sessions of VR training utilizing 3D video games. The intervention revealed that the EG had a significant positive effect on all static balance measures, as well as lower limb strength. The results are presented in Table 4.

Sato, et al. [44] conducted a randomized controlled trial on 54 older adults with the aim of testing the effects of playing an exergame on exercise function in older subjects, analyzing its influence on balance, gait, and muscle strength. The EG performed three 40–60-min sessions per week for 8 weeks of exergame therapy using Kinect and Kinect SDK version 1.5 and Unity version 3.4.2, while the CG continued their normal daily activities. Within-group statistical analysis revealed improvements in EG for balance, stability, and lower limb strength. The results are shown in Table 4.

#### 3.4.2. Virtual Reality Therapy Compared to Balance Training

Six studies [46,47,50,54,56,57] compared balance training using VR devices with conventional balance training in older adults. The main characteristics and results of the studies included in this section are presented in Table 5.

Fu, A.S. et al. [47] conducted a randomized controlled trial to investigate the effect of interactive exergaming training on balance control, fall risk factors, and fall incidence in 60 frail elderly nursing home residents. The EG performed three 60-min sessions per week for 6 weeks of balance training using a Nintendo Wii Fit balance board, while the CG performed a conventional balance training program. Significant variations were noted both between and within the groups. Although both groups demonstrated improvements in quadriceps strength, reaction time, postural sway, and risk of falls based on within-group analysis, the experimental group displayed significantly greater results compared to those who received conventional balance training, as indicated by the between-group analysis (Table 5). Additionally, the intervention group had an overall incidence of falls of 0.54 per person per year (range 0–1), which was lower than the control group’s incidence of 1.52 per person per year (range 0–3).

Khushnood, K. et al. [54] developed an RCT with the aim of detecting changes in gait and balance after using the Wii Fit gaming platform in older adults. Eighty-three older adults with a mean age of 65.75 years were divided into two groups. The EG performed a 30-min balance training program on the Wii Fit gaming platform, while the CG performed a program of balance training exercises. In terms of balance, although the results showed no statistically significant differences in between-group comparisons (*p* = 0.22), both groups showed significant improvements between pre- and post-intervention assessments (EG [*p* < 0.001] and CG [*p* < 0.001]). With respect to gait, comparisons between groups showed statistically significant improvements in favor of EG (variability [*p* < 0.001], alertness [*p* < 0.001], sway [*p* < 0.001], hip range of motion [*p* < 0.001], and leg-shoulder synchrony [*p* < 0.001]), although both groups showed statistically significant improvements between pre- and post-treatment assessments for all gait parameters (sway [EG [*p* < 0.001] and CG [*p* < 0.004]], foot contact [EG [*p* < 0.001] and CG [*p* < 0.001]], hip range of motion [EG [*p* < 0.001] and CG [*p* < 0.001]], and leg-shoulder synchrony [EG [*p* < 0.001] and CG [*p* < 0.001]], except for variability (EG [*p* < 0.001] and CG [*p* = 0.66]) and vigilance (EG [*p* < 0.001] and CG [*p* < 0.55]).

Lima-Rebêlo, F. et al. [56] compared a balance exercise protocol and a walking-with-obstacles control intervention to an immersive VR exercise program in a group of 37 older adults with an average age of 70.24 years. The immersive VR program consisted of sixteen twice-a-week sessions, during which participants played four games. In this instance, intragroup analysis revealed statistically significant changes in both groups for functional balance, gait, and sensory interaction variables (DGI, CTSIB, and FRT). Furthermore, only the experimental group exhibited increased mobility (TUG) and decreased dizziness (DHI). The gains in functional balance were sustained after two months, but gains in functional reach decreased for both groups.

Strunz, T. et al. [57] conducted a randomized controlled trial to demonstrate the effects of an interactive balance game program on dynamic balance control compared to a physical therapy balance exercise program. The EG performed two 30-min sessions per week for 8 weeks of dynamic balance exercises coupled with computer games, while the CG performed a physical therapy program of balance exercises. In the between-group analysis, the experimental group showed significantly greater improvements in change scores for static balance and fear of falling compared to the control group. The results are displayed in Table 5.

Tsang, W.W.N. et al. [50] compared balance training using Wii Fit with conventional balance training in a group of older adults. They conducted an RCT on seventy-nine older adults with a mean age of 82.15 years who were randomly assigned to two groups. The EG performed a balance training program using Wii Fit, while the CG performed a conventional balance training program. The results of the between-group comparison showed statistically significant improvements in favor of the EG in balance (*p* < 0.001) and in two of the four gait parameters (Table 5). Furthermore, statistically significant improvements were observed in both groups for both static balance (EG [*p* < 0.01] and CG [*p* < 0.01]) and dynamic balance (EG [*p* < 0.01] and CG [*p* = 0.01]) when comparing pre- and post-intervention measurements.

Yeşilyaprak, S. et al. [46] conducted an RCT to investigate the effects of VR-based balance training on balance and fall risk compared to conventional balance training. Eighteen older adults with a mean age of 71.6 years were randomized into two groups. The EG received a balance training program using the NIRVANA Interactive Virtual Reality System, while the CG received a conventional balance training program. Although the between-group analysis did not show statistically significant differences, the within-group analysis showed statistically significant improvements in both groups in both static and dynamic balance when comparing the pre- and post-intervention assessments (Table 5).

#### 3.4.3. Virtual Reality Therapy Compared to Physical Exercise

Finally, five studies [41,43,44,49] included in this systematic review aimed to analyze the impact of VR training on balance training versus traditional physical exercise in older adults. The main characteristics and results of the studies included in this section are presented in Table 6.

Karahan, A.Y. et al. [41] conducted a randomized controlled trial to demonstrate the effects of EG using the Xbox 360 Kinect game console on balance, functional mobility, and quality of life in geriatrics and to compare the effects of EG with those achieved in the home exercise program. The EG performed five 30-min sessions per week for 6 weeks of balance training using a game set consisting of the Xbox 360 Kinect game, while the CG performed the home exercise program. Although within-group analysis showed that balance scores significantly improved in both groups (all BBS parameters in both groups [*p* < 0.05]), TUG and SF-36 scores only improved in the EG group. In addition, the analysis between the groups showed a significant improvement in favor of the EG for the BBS (Table 6).

Lee, K. et al. [48] evaluated the effect of VR gait training on balance by conducting a RCT on 56 older adults with a mean age of 80.24 years. The participants were randomly assigned to two groups. The EG underwent gait training with Virtual Reality (VR), while the CG underwent treadmill gait training without VR. On comparing both groups, the results indicated that the EG had statistically significant improvements in single-leg balance (t = 6.240, *p* < 0.001) and dynamic balance (t = 3.339, *p* = 0.002). Additionally, the EG showed significant improvements in the spatial parameters of gait as compared to the CG (the results are detailed in Table 6). Significant improvements in dynamic balance (t = 4.600; *p* < 0.001) and walking speed (t = 3.452; *p* = 0.002) were observed in the experimental group (EG), as compared to pre- and post-intervention evaluations. Both groups showed improvements in spatial parameters of gait, including static stride length (EG [t = 4.875; *p* < 0.001]; CG [t = 3.134; *p* = 0.004]), and step length (EG [t = 4.875; *p* < 0.001]; CG [t = 3.134; *p* = 0.004]).

Park, J. et al. [43] conducted a RCT to assess the efficacy of a 3D virtual reality program utilizing Kayak exercises to enhance balance, cognitive function, and muscle strength among older adults. The study population consisted of seventy-two older adults with a mean age of 73.54 years who were assigned randomly to two groups. Both groups were engaged in a traditional exercise program, while the intervention group also performed a 20-min virtual reality training in Kayak making use of a 3D projector and 3D images. The results indicated that the EG demonstrated significant differences in all balance parameters (Table 6) when compared to the CG. Significant improvements were also noted in both groups when comparing the pre- and post-intervention assessments for all balance variables assessed with eyes closed, upper limb strength, and cognitive function.

In the study of Ribeiro-Bacha, J.M. et al. [49], the objective was to compare the effects of Kinect Adventures games and a conventional physical therapy training program on balance, gait, cognition, and cardiorespiratory function in older adults. To achieve this goal, they conducted a RCT with 46 participants who were randomly assigned to either the EG, which played Xbox Kinect Adventures games, or the CG, which underwent a physical therapy training program in small groups under the supervision of a physical therapist. Although there were no statistically significant differences between both groups, both the EG (*p* < 0.005) and CG (*p* < 0.001) showed statistically significant intragroup improvements at the end of treatment. The improvements were consistently higher in the EG (MD of the EG = 2.26; MD of the CG = 1.34). Statistically significant intragroup improvements in gait were found in both the EG and CG (Table 6) and these improvements were maintained over time. Additionally, the CG showed a statistically significant improvement in cardiorespiratory fitness in the post-treatment evaluation, which persisted at follow-up (*p* < 0.001). In contrast, the EG only demonstrated a statistically significant improvement in the follow-up evaluation (*p* < 0.05).

Finally, Yang, C.M. et al. [45] conducted a randomized controlled trial to investigate the impact of using the Kinect VR device (Microsoft, Washington, DC, United States) for balance training, in comparison to conventional physical exercise, among 20 older adults. The experimental group engaged in exercises utilizing the Microsoft Kinect for Xbox 360 with “Your Shape: Fitness Evolved II” software, while the control group adhered to a physical exercise program specially designed to prevent falls in the elderly. The dynamic balance of the EG showed superior results when compared to the CG. Although there were no significant differences found between the groups, both groups showed statistically significant intragroup differences in the 30-s CST (EG [z = −2. 818; *p* = 0.005]), the FRT (EG [z = −2.803; *p* = 0.005]; CG [z = −2.803; *p* = 0.005]), and OLS with eyes open (EG [z = −2.803; *p* = 0.005]; CG [z = −1.988; *p* = 0.047]). The results were solely noted in the experimental group during the one-leg standing test with eyes closed and the timed up and go test. The results are presented in Table 6.

## 4. Discussion

There are numerous diseases, deficiencies, and degenerative changes that affect physical and cognitive functions associated with aging. Among others, deterioration of vision, gait, muscle strength, balance, and cognition can be observed [58], leading to a decrease in physical activity, resulting in a deconditioning cycle [1] that usually ends with the appearance of balance disorders [4]. All of these changes usually have a major impact on daily life, leading to mobility problems, which in turn limit independence and psychological well-being, in addition to having a major economic and health impact [1]. All of the above, together with the emergence of new and promising approaches such as VR, which is currently booming, led us to gather the most recent evidence on therapy with VR systems in populations of older adults, with the aim of evaluating its effectiveness on balance and gait.

Older people may benefit from VR therapy due to its stimulation of balance, both static and dynamic [59]. Consistent with previous findings [60,61,62], in our review, when comparing the effect of VR therapy to minimal intervention or usual care, improvements in static and dynamic balance, gait, stability, fear of falling, risk of falling, strength, and quality of life were observed in patients who completed VR-based exercise programs [39,40,42,44,51,52,53,55]. These results may be based on VR training promoting upper and lower limb mobility and strength and trunk control, stimulating joint proprioceptive referents, and improving postural adjustment ability [63]. These factors reduce the risk of falling and generate an increase in confidence, reducing the fear of falling and improving the performance of activities of daily living. As a result, quality of life is improved.

On the other hand, and in contrast to the above, Adcock M. et al. [38] found no improvement in balance or gait. The lack of results is most likely due to the excessive duration of treatment (4 months). One of the most important factors in achieving good results in terms of balance and fall prevention is adherence to treatment [22]. Considering that this is a population that may be occasionally dependent, the difficulties they usually have with technology, and the fact that the treatment sessions take place at home, such a long treatment could lead to a loss of interest on the part of the participants and therefore a reduction in adherence to treatment.

Conventional balance training has been widely shown to be effective in both treating balance disorders [64] and reducing the rate of falls in the elderly population [65]. In the present review, VR-based therapy was found to have better results than conventional balance training in treating balance disorders and gait. The sense of balance is based on the integration of inputs from the vestibular, visual, and somatosensory systems [66], so the treatment of its possible alterations should focus on addressing the aforementioned systems. In this sense, VR-based balance training involves all three [67,68,69]. VR therapy involves performing dynamic activities and multidirectional head movements that cause linear accelerations and decelerations, as well as changes in their gravitational inertia, which would stimulate the vestibular system [46]. At the same time, VR provides multiple visual challenges [69,70], which are a great stimulus for treating balance disorders. This may be why, despite the efficacy of both therapies in treating balance and gait disorders, VR therapy demonstrates superior outcomes.

Loss of lower extremity strength and impaired balance due to physical inactivity can predict falls among non-institutionalized elderly individuals. This review demonstrates that both virtual reality therapy and physical exercise effectively improve static and dynamic balance, gait, and lower limb strength. In accordance with previous studies [71], it is noteworthy that the patients who underwent VR therapy showed greater improvement compared to those who engaged in physical exercise. This increase in muscle strength in both groups is consistent with lower extremity training being a key component of balance therapy, as it has been observed to be correlated with improved functional activity performance [72]. The enhancement in functional activity performance, resulting from postural control and position changes during video game interaction in patients undergoing VR therapy, can potentially enhance dynamic balance, boost muscle strength, and stimulate the role of mirror neurons [27]. Loss of lower extremity strength and impaired balance due to physical inactivity can predict falls among non-institutionalized elderly individuals. This review demonstrates that both virtual reality therapy and physical exercise effectively improve static and dynamic balance, gait, and lower limb strength. In accordance with previous studies [71], it is noteworthy that the patients who underwent VR therapy showed greater improvement compared to those who engaged in physical exercise. The enhancement of muscle strength in both groups aligns with the plan in response to the lower limb training, as it is a cornerstone of balance therapy’s physical exercise strength. Moreover, it has been observed that the enhancement of muscle strength correlates with its improvement [72]. The enhancement in functional activity performance, resulting from postural control and position changes during video game interaction in patients undergoing VR therapy [27], can potentially enhance dynamic balance, boost muscle strength, and stimulate the role of mirror neurons. In addition, static balance and postural control may be significantly improved by the physiological demands of VR therapy. Increasing knee proprioception through the repeated shifting of body weight between both legs, a necessary component of VR therapy [73], can lead to significant progress in static balance. While both therapies showed improvement, VR therapy produced better results possibly due to the precision with which its systems capture movement and the intensive visual feedback [74,75]. This, in conjunction with targeted movement tasks, enables more efficient and correct movement and body posture, owing to the continuous visual feedback patients receive from the device.

Despite obtaining relevant information about the effectiveness of VR on balance and gait in older adults, this systematic review has limitations. Heterogeneity was observed in terms of intervention protocols in VR therapy, including the type of exercise, the number of repetitions, the series, the duration of the session, and intervention. Additionally, evaluation tools of different variables were varied, thus limiting the external validity of the obtained results. However, it is important to note the methodological quality of the studies included in this review, indicating the reliability and stability of the results obtained. Given the nature of the research, it is worthwhile to further investigate with the goal of achieving agreement in terms of the parameters of the virtual reality intervention.

## 5. Conclusions

Based on the systematic review, it can be concluded that VR therapy is more effective than minimal intervention or usual care in enhancing static balance, dynamic balance, and gait in healthy older adults. Moreover, compared to conventional balance training, VR therapy exhibits superior outcomes for balance and gait disorders in this population. VR therapy is superior to physical exercise for older adults in enhancing their static and dynamic balance, gait, and lower limb strength. Nonetheless, both methods proved effective.

## Figures and Tables

**Table 1:** Search strategy for each database.

Database	Search Strategy
PubMed	(aged[mh] or aged[tiab] or aged, 80 and over[mh] or aged, 80 and over[tiab] or frail elderly[mh] or frail elderly[tiab] or old* adult*[tiab] or elderly[tiab] or ancient*[tiab]) AND (virtual reality[mh] OR virtual reality[tiab] OR virtual reality exposure therapy[mh] OR virtual reality exposure therapy[tiab] OR exergam*[tiab] or video games [mh] or video-gam*[tiab]) AND (postural balance[mh] or postural balance[tiab] or balance[mh] or balance[tiab] or balance*[tiab] or balance disorder*[tiab] or equilibrium[tiab])
SCOPUS	[TITLE-ABS-KEY (“aged” OR “elderly” OR “older adult”) AND TITLE-ABS-KEY (“virtual reality” OR “video games” OR “exergames”) AND TITLE-ABS-KEY (“postural balance” OR “balance” OR “balance disorders”)]
Web of Science	TOPIC: (*aged* OR *elderly* OR *frail elderly* OR *older adult*) AND TOPIC: (*virtual reality* OR *exergame* OR *video games*) AND TOPIC: (*postural balance* OR *balance* OR *balance disorders*)
PEDro	aged AND virtual reality AND balance aged AND exergame AND balance aged AND video games AND balance older adults AND virtual reality AND balance older adults AND exergame AND balance older adults AND video game AND balance elderly AND virtual reality AND balance elderly AND exergame AND balance elderly AND video games AND balance

**Table 2:** PEDro scores for assessing the methodological quality and risk of bias of the studies included in the systematic review.

Study	I1	I2	I3	I4	I5	I6	I7	I8	I9	I10	I11	Total	Quality
**Adcock et al., 2021 [38]**	Y	Y	Y	Y	N	N	N	Y	N	Y	Y	6/10	Good
**Benítez-Lugo et al., 2022 [51]**	Y	Y	Y	Y	Y	Y	Y	Y	N	Y	N	8/10	Good
**Campo-Prieto et al., 2022 [39]**	Y	Y	Y	Y	N	N	N	Y	Y	Y	N	6/10	Good
**Delbroek et al., 2017 [40]**	Y	Y	Y	Y	N	N	Y	Y	Y	N	N	6/10	Good
**Fu et al., 2015 [47]**	Y	Y	Y	Y	N	N	Y	Y	Y	Y	N	7/10	Good
**Gallardo-Meza et al., 2022 [52]**	Y	Y	Y	Y	N	Y	Y	Y	N	Y	Y	8/10	Good
**Gomes et al., 2018 [53]**	Y	Y	Y	Y	N	N	Y	Y	Y	Y	Y	8/10	Good
**Karahan et al., 2015 [41]**	Y	Y	Y	Y	N	N	Y	Y	N	Y	N	6/10	Good
**Khusnood, K. et al., 2021 [54]**	Y	Y	Y	N	Y	Y	Y	Y	N	Y	Y	8/10	Good
**Kim, S.H. et al., 2022 [55]**	Y	Y	Y	Y	N	N	Y	Y	Y	Y	Y	8/10	Good
**Lee, K. et al., 2021 [48]**	Y	Y	Y	Y	N	N	Y	Y	Y	Y	N	7/10	Good
**Lee, Y. et al., 2017 [42]**	Y	Y	Y	Y	N	N	Y	Y	N	Y	N	6/10	Good
**Lima-Revêlo et al., 2021 [56]**	Y	Y	Y	Y	Y	Y	Y	Y	N	Y	N	8/10	Good
**Park et al., 2016 [43]**	Y	Y	Y	Y	N	N	N	Y	Y	Y	N	6/10	Good
**Ribeiro-Bacha et al., 2018 [49]**	Y	Y	Y	Y	N	N	Y	Y	N	Y	Y	7/10	Good
**Sato et al., 2015 [44]**	Y	Y	Y	Y	N	N	Y	Y	N	Y	N	6/10	Good
**Szturm et al., 2011 [57]**	Y	Y	Y	Y	N	N	Y	Y	Y	Y	Y	8/10	Good
**Tsang et al., 2020 [50]**	Y	Y	N	Y	N	N	Y	Y	Y	Y	Y	7/10	Good
**Yang et al., 2020 [45]**	Y	Y	N	Y	N	N	Y	Y	Y	Y	N	6/10	Good
**Yeşilyaprak et al., 2016 [46]**	Y	Y	Y	Y	N	N	N	Y	N	Y	Y	6/10	Good

Abbreviations: I1: Eligibility criteria; I2: Randomized distribution; I3: Allocation concealment; I4: Comparability at baseline; I5: Blinded subjects; I6: Blinded therapists; I7: Blinded assessors; I8: Adequate monitoring; I9: Intention-to-treat analysis; I10: Between-groups comparison; I11: point estimation and variability; Y, Yes; N, No. Note: Item 1 does not contribute to the final score. Note: Score confirmed in PEDro webpage.

**Table 3:** Characteristics of the studies included in the systematic review.

Study	Participants	EG Sample Characteristics	CG Sample Characteristics
**Adcock, M. et al., 2021 [38]** **Design:** RCT	31 Older adults (16F/15M)	15 Subjects (77 ± 6.4 year; 10F/5M)	16 patients (70.9 ± 5.0 year; 6F/10M)
**Benítez-Lugo, M.L. et al., 2022 [51]** **Design:** RCT	46 Older adults (32F/14M)	26 Subjects (72.19 ± 4.79 year; 20F/6M)	20 Subjects (73.00 ± 7.4 year; 12F/8M)
**Campo-Prieto, P. et al., 2022 [39]** **Design:** RCT	24 Older adults (21F/3M)	13 Subjects (85.08 ± 8.48 year; 11F/2M)	11 Subjects (84.82 ± 8.10 year; 10F/1M)
**Delbroek, T. et al., 2017 [40]** **Design:** RCT	20 Older adults (13F/5M)	10 Subjects (86.9 ± 5.6 year; 2F/8M)	10 Subjects (87.5 ± 6.6 year; 5F/5M)
**Fu, A.S. et al., 2015 [47]** **Design:** RCT	60 Older adults (39F/21M)	30 Subjects (82.4 ± 3.8 year; 20F/10M)	30 Subjects (82.3 ± 4.3 year; 19F/11M)
**Gallardo-Meza, C. et al., 2022 [52]** **Design:** RCT	72 Older women	35 Subjects (69.2 ± 3.3 year; 35F/0M)	37 Subjects (68.1 ± 3.7year; 37F/0M)
**Gomes, G.C.V. et al., 2018 [53]** **Design:** RCT	30 Older adults (Gender not reported)	15 Subjects (83 ± 5.87 year; Gender not reported)	15 Subjects (85 ± 6.19 year; Gender not reported)
**Karahan, A.Y. et al., 2015 [41]** **Design:** RCT	90 Older adults (39F/21M)	48 Subjects (71.3 ± 6.1 year; 21F/27M)	42 Subjects (71.5 ± 4.7 year; 18F/24M)
**Khusnood, K. et al., 2021 [54]** **Design:** RCT	83 Older adults (Gender not reported)	42 Subjects (65 ± 3.0 year; Gender not reported)	41 Subjects (66.5 ± 4.6 year; Gender not reported)
**Kim, S.H. et al., 2022 [55]** **Design:** RCT	34 Older adults (39F/21M)	12 Subjects (75.75 ± 10.15 year; 21F/27M)	12 Subjects (80.75 ± 6.03 year; 21F/27M)
			10 Subjects (83.10 ± 5.24 year; 21F/27M)
**Lee, K. et al., 2021 [48]** **Design:** RCT	56 Older adults (39F/21M)	28 Subjects (81.01 ± 6.89 year; 12F/16M)	28 Subjects (79.47 ± 6.15 year; 13F/15M)
**Lee, Y. et al., 2017 [42]** **Design:** RCT	40 Older adults (Gender not reported)	21 Subjects (76.15 ± 4.55 year; 12F/9M)	19 Subjects (75.71 ± 4.91 year; 11F/8M)
**Lima-Revêlo, F. et al., 2021 [56]** **Design:** RCT	37 Older adults (31F/6M)	17 Subjects (69.25 ± 5.67 year; 16F/4M)	20 Subjects (71.41 ± 5.94 year; 15F/2M)
**Park, J. et al., 2016 [43]** **Design:** RCT	72 Older adults (68F/4M)	36 Subjects (72.97 ± 2.98 year; 33F/3M)	36 Subjects (74.11 ± 2.88 year; 35F/1M)
**Ribeiro-Bacha, J.M. et al., 2018 [49]** **Design:** RCT	46 Older adults (34F/12M)	23 Subjects (66.5 year; 19F/4M)	23 Subjects (71.0 year; 15F/8M)
**Sato, K. et al., 2015 [44]** **Design:** RCT	54 Older adults (43F/11M)	28 Subjects (70.07 ± 5.35 year; 22F/6M)	26 Subjects (68.50 ± 5.47 year; 21F/5M)
**Szturm, T. et al., 2011 [57]** **Design:** RCT	27 Older adults (19F/8M)	13 Subjects (80.5 ± 6 year; 10F/3M)	14 Subjects (81 ± 7 year; 9F/5M)
**Tsang, W.W.N. et al., 2020 [50]** **Design:** RCT	79 Older adults (48F/31M)	39 Subjects (82.3 ± 3.8 year; 23F/16M)	40 Subjects (82.0 ± 4.3 year; 25F/15M)
**Yang, C.M. et al., 2020 [45]** **Design:** RCT	20 Older adults (18F/2M)	10 Subjects (69.71 year; 9F/1M)	10 Subjects (67.54 year; 9F/1M)
**Yeşilyaprak, S.S. et al., 2016 [46]** **Design:** RCT	18 Older adults (5F/13M)	7 Subjects (70.1 ± 4.0 year; 3F/4M)	11 Subjects (73.1 ± 4.5 year; 2F/9M)

Abbreviations. EG: Experimental Group; CG: Control Group; RCT: Randomized Clinical Trial; F: Female; M: Male.

**Table 4:** Characteristics and results of studies comparing virtual reality therapy to usual care or minimal intervention.

Study	EG Intervention	CG Intervention	Assessments	Variables	Tests	Outcomes
**Adcock, M. et al., 2021 [38]**	48 sessions. Three 30–40 min sessions per week for 16 weeks. Strength, balance, and cognitive training through Active@ home exergames.	Normal daily activity	Patients were assessed preintervention and after intervention.	Balance	SPPB	At post treatment improvements in WMS-R forward span score (F = 8.504, *p* = 0.004, η_p_^2^ = 0.04) and WMS-R backward span score (F = 5.872, *p* = 0.015, η_p_^2^ = 0.02) and no significant improvements in balance (*p* > 0.05) or gait (*p* > 0.05) at post treatment were observed.
				Gait	Physilog^®^ 5.	
				Lower body strength and aerobic endurance	SFT	
				Cognitive Functions	VST	
					WMS-R	
					TMT	
**Benítez-Lugo, M.L. et al., 2022 [51]**	16 sessions. Two 30 min sessions per week for 8 weeks. Wii Fit exercise program for Nintendo Wii, using the Wii Balance Board platform.	Usual care (memory workshops and joint mobility workshops in the center).	Patients were assessed pre intervention and after intervention	Balance	BBS	EG showed greater improvements in terms of balance, gait, and fall risk (BBS [*p* = 0.001]; TUG [*p* = 0.001]; Tinetti [*p* = 0.001]), attention, and cognitive status (Oddball [*p* = 0.003]; MMSE [*p* = 0.025]). [*p* = 0.001])
					Tinetti test	
				Gait	TUG	
					Tinetti test	
				Risk of fall	Tinetti test	
				Attention	ANT-ELDERLY	
					Oddball Test	
				Cognitive Status	MMSE	
**Campo-Prieto, P. et al., 2022 [39]**	30 sessions. Three 6 min sessions per week for 10 weeks. Exercise-based boxing in a virtual gym space while wearing immersive virtual reality goggles.	Usual care (memory workshops and occupational therapy).	Patients were assessed pre intervention, after intervention, and at 14 weeks.	Balance	Tinetti test	EG showed greater improvements in terms of balance, gait, and lower limb function, both at post treatment (Tinetti balance [*p* < 0.005]; Tinetti gait [*p* < 0.005]; Tinetti total [*p* < 0.005]; TUG [*p* < 0.005]; FTSTS [*p* < 0.005]) and at follow-up (Tinetti balance [*p* < 0.005]; Tinetti gait [*p* < 0.005]; Tinetti total [*p* < 0.005].
				Gait	Tinetti test	
				Lower limb function	TUG	
					FTSTS	
				Handgrip Strength	Dynamometer	
				QoL	SF-12	
**Delbroek, T. et al., 2017 [40]**	12 sessions. Two 18–30 min sessions per week for 6 weeks. Training program with Bio Rescue. This is a virtual reality device for cognitive-motor training of dual tasks.	Usual care in the nursing home if applicable.	Patients were assessed pre intervention and after intervention	Balance	Tinetti test	EG showed greater improvements in three gait domains, including a reduction in total ambulation time (*p* = 0.02), sitting transition (*p* = 0.02), and gait time before turning (*p* = 0.02).
				Gait	TUG	
				Cognitive Functions	MoCA	
**Gallardo-Meza, C. et al., 2022 [52]**	Eight sessions. Two sessions per week for 4 weeks. Active exergames training program (Nintendo Wii, including the Wii Fit Plus, the Wii Balance board, and the Wii Nunchuk).	Habitual weekly physical activity levels.	Patients were assessed pre intervention and after intervention	Balance	One-leg stance test	EG showed greater improvements in strength, with a Δ75.5% increase (d = 0.89) in right leg static balance, a Δ33.7% increase (d = 0.57) in left leg static balance, a Δ14.8% increase (d = 0.85) in timed stand-and-walk test, and a Δ83.8% increase (d = 1.62) in five-repetition sit-to-stand test.
				Gait	TUG	
				Lower limb strength	FTSTS velocity test	
**Gomes, G.C.V. et al., 2018 [53]**	14 sessions. Two 50 min sessions per week for 7 weeks. Exercise program with Nintendo Wii fit plus adapted to the needs, supervised by a physiotherapist.	World Health Organization guidelines described the benefits and risks of physical activity.	Patients were assessed pre intervention, after intervention, and at 1 month after treatment.	Balance	Mini-BEST test	EG showed greater improvements in static balance, measured by the Mini-BEST test (F = 6.060; *p* = 0.004; ES = 0.016), and gait, measured by the FGA (F = 3.794; *p* = 0.028; ES = 0.014), than the CG.
				Gait	FGA	
				Fear of Falling	FES-I	
				Cognitive functions	MoCA	
				Mood	GDS-15	
**Kim, S.H. et al., 2022 [55]**	18 sessions. Three 30 min sessions per week for 6 weeks. Wii Fit game program.	No intervention.	Patients were assessed pre intervention, after intervention, and at two weeks after treatment.	Balance	Personal measurement	EG showed greater improvements in OE balance sensation compared to the CG after treatment (*p* = 0.020; d = 1.08).
				Body center movement area	Gaitview AFA-50 system	
		18 sessions. Three 20 min sessions per week for 6 weeks. Motor imagery training		Fear of falling	Tinetti test	
					BBS	
**Lee, Y. et al., 2017 [42]**	Virtual reality training with 3D video games.	Fall prevention education program.	Patients were assessed preintervention and after-intervention.	Static Balance	Stabilometric platform	EG showed greater improvements in medio-lateral sway (OE [*p* = 0.025], CE [*p* = 0.010]), anterior-posterior sway (OE [*p* = 0.018], CE [*p* = 0.017]), and travel speed (OE [*p* = 0.011], CE [*p* = 0.010]). In monopodal balance, significant improvements in antero-posterior sway (*p* = 0.017) and velocity of displacement (*p* = 0.010), for both OE (*p* = 0.008) and CE (*p* = 0.035), dynamic balance (BBS [*p* < 0.001], FRT [*p* < 0.001], and TUG [*p* < 0.001]) and lower limb strength (*p* < 0.001).
					One-leg stance test	
				Dynamic Balance	BBS	
					TUG	
					Functional reach test	
				Lower body strength	FTSTS	
**Sato, K. et al., 2015 [44]**	Therapy with Kinect and Kinect SDK version 1.5 and Unity version 3.4.2.	Normal daily activity.	Patients were assessed pre intervention and after intervention.	Balance	BBS	Within-group statistical analysis revealed improvements in EG for balance (*p* < 0.01), stability(*p* < 0.01), and lower limb strength (*p* < 0.01), while CG showed no differences between pre- and post-assessments. In addition, gait analysis showed a decrease in double stance time in EG (*p* = 0.04).
				Gait	3D motion analysis system Cortex 2	
				Stability	FRT	
				Lower limb strength	30-s Chair Stand Test	

Abbreviations. ANT-ELDERLY: Attention Network Test-ELDERLY; BBS: Berg Balance Test; CG: Control Group; EG: Experimental Group; FES-I: Fall Efficacy Scale International; FGA: Functional Gait Assessment; FRT: Functional Reach Test; FTSTS: Five Time Sit to Stand Test; GDS: Geriatrics Depression Scale; MMSE: Mini Mental State Examination; MoCA: Montreal Cognitive Assessment; QoL: Quality of Life; SF-12: 12 Items Short form Survey; SFT: Senior Fitness Test; SPPB: Short Physical Performance Battery; TMT: Trail Making Test; TUG: Timed- UP and Go test; VST: Victoria Stroop Test; WMS-R: Wechsler Memory Scale-Revised.

**Table 5:** Characteristics and results of studies comparing virtual reality to balance training.

Study	EG Intervention	CG Intervention	Assessments	Variable	Test	Outcomes
**Fu, A.S. et al., 2015 [47]**	18 sessions. Three 60-min sessions per week for 6 weeks. Balance training using a Nintendo’s Wii Fit balance board.	18 sessions. Three 60-min sessions per week for 6 weeks. Conventional balance training.	Patients were assessed pre intervention and after intervention.	Falls	Fall incidence	EG showed greater improvements compared to CG in terms of muscle strength (*p* < 0.001), faster reaction times (*p* < 0.001), and less body sway (*p* = 0.013).
				Risk of fall	PPA	
				Postural Sway	Sway meter recording displacements of the body	
				Quadriceps Strength	Kilograms	
**Khusnood, K. et al., 2021 [54]**	16 sessions. Two 30-min sessions per week for 8 weeks. Exergaming with the Wii fit.	16 sessions. Twice a week for 8 weeks. Balance exercise.	Patients were assessed pre intervention, after intervention, and at 8 weeks after treatment.	Balance	BBS	EG showed greater improvements compared to CG in terms of gait (variability [*p* < 0.001], alertness [*p* < 0.001], sway [*p* < 0.001], hip range of motion [*p* < 0.001], and leg-shoulder synchrony [*p* < 0.001]).
				Gait	GARS-M	
**Lima-Revêlo, F. et al., 2021 [56]**	16 sessions. Two 60-min sessions per week for 6 weeks. Immersive Virtual Reality program through Oculus rift device (Menlo Park, CA, USA).	16 sessions. Two 60-min sessions per week for 6 weeks. Balance exercise.	Patients were assessed pre intervention, after intervention, and at 1 month after treatment.	Static Balance	CTSIB	Only the experimental group exhibited increased mobility (TUG) and decreased dizziness (DHI).
				Gait	TUG	
					DGI	
				Stability	FRT	
				Fear of Falling	FES-I	
				Dizziness	DHI	
**Szturm, T. et al., 2011 [57]**	16 sessions. Two 30-min sessions per week for 8 weeks. Dynamic balance exercise coupled with computer games.	16 sessions. Two 30-min sessions per week for 8 weeks. Conventional balance training.	Patients were assessed pre intervention and after intervention.	Static Balance	BBS	EG showed greater improvements compared to CG in change scores for the BBS (*p* = 0.001), LOB counts (*p* = 0.007), and ABC (*p* = 0.020) compared to the control group.
				Dynamic Balance	CTSIB	
				Gait	TUG	
					GaitRite Instrumented carpet System	
				Fear of Falling	ABC	
**Tsang, W.W.N. et al., 2020 [50]**	18 sessions. Three 60-min sessions per week for 6 weeks. Wii Fit balance training.	18 sessions. Three 60-min sessions per week for 6 weeks. Conventional balance training.	Patients were assessed pre intervention and after intervention.	Static Balance	BBS	EG showed greater improvements compared to CG in balance (*p* < 0.001), end point of excursion (anterior [*p* < 0.001], posterior [*p* = 0.001], left [*p* = 0.048], and right [*p* = 0.007]) and maximum excursion (anterior [*p* < 0.001], posterior [*p* < 0.001], left [*p* = 0.011], and right [*p* = 0.001]).
				Dynamic Balance	TUG	
				Stability limits	Limits of stability test	
**Yeşilyaprak, S.S. et al., 2016 [46]**	18 sessions. Three 60-min sessions per week for 6 weeks. Balance training with the NIRVANA Interactive Virtual Reality System.	18 sessions. Three 60-min sessions per week for 6 weeks. Conventional balance training.	Patients were assessed pre intervention and after intervention.	Static Balance	BBS	The within-group analysis showed improvements in both groups in both static (EG BBS *p* < 0.01; CG BBS *p* < 0.01) and dynamic balance (EG TUG *p* = 0.01; CG TUG *p* = 0.04).
					One-leg stance test	
					Tandem stance test	
				Dynamic Balance	TUG	
				Fear of Falling	FES-I	

Abbreviations. ABC: Balance Confidence Scale; BBS: Berg Balance Test; CG: Control Group; CTSIB: Clinical Test of Sensory Interaction and Balance; DGI: Dynamic Gait Index; DHI: Dizziness Handicap Inventory; EG: Experimental Group; FES-I: Fall Efficacy Scale International; FRT: Functional Reach Test; GARS-M: Gait abnormality Rating Scale-Modified; PPA: Physiological Profile Assessment; TUG: Timed Up and Go test.

**Table 6:** Characteristics and results of studies comparing virtual reality therapy compared to physical exercise.

Study	EG Intervention	CG Intervention	Assessments	Variables	Tests	Outcomes
**Karahan, A.Y. et al., 2015 [41]**	30 sessions. Five 30-min sessions per week for 6 weeks. Game set comprising the Xbox 360 Kinect game (Kinect Adventures, Kinect Sports, and Kinect Sports Season two).	30 sessions. Five 30-min sessions per week for 6 weeks. Balance training.	Patients were assessed before and after the intervention.	Balance	BBS	EG showed greater improvements compared to CG in BBS (*p* < 0.001). In addition, within-group analysis showed EG group improvements in TUG and SF-36 (all *p* < 0.05)
				Gait	TUG	
				QoL	SF-36	
**Lee, K. et al., 2021 [48]**	20 sessions. Five 50-min sessions per week for 4 weeks. Virtual reality gait training.	20 sessions. Five 50-min sessions per week for 4 weeks. Standard treadmill training without virtual reality.	Patients were assessed before and after the intervention.	Balance	One-leg stance test	EG showed greater improvements compared to CG in single-leg balance (t = 6.240, *p* < 0.001) and dynamic balance (t = 3.339, *p* = 0.002), static stride (t = 2.136, *p* = 0.037), step length (t = 2.136, *p* = 0.037), and step width (t = 2.364, *p* = 0.022).
					BBS	
				Gait	TUG	
					OptoGait	
				Stability	FRT	
**Park, J. et al., 2016 [43]**	12 sessions. Two 50-min sessions per week for 6 weeks. 30 min of conventional exercise and 20 min of virtual training in Kayak (using a 3-D projector and 3-D images).	12 sessions. Two 30-min sessions per week for 6 weeks. Conventional exercise.	Patients were assessed before and after the intervention.	Balance	Stabilometric platform	EG showed greater improvements compared to CG in all balance parameters, including OE (t = 4.367; *p* = 0.000) and CE (t = 4. 367; *p* = 0.000), upper limb strength (ACT; t = 6.896; *p* = 0.000), grip strength for the right hand (t = 4.367; *p* = 0.000) and left hand (t = 5.836; *p* = 0.000), and cognitive function (t = 5.475; *p* = 0.000).
				Upper limb muscle strength	Dynamometer	
					ACT	
				Cognitive Functions	MoCA	
**Ribeiro-Bacha, J.M. et al., 2018 [49]**	14 sessions. Two 60-min sessions per week for 7 weeks. Therapy with Xbox Kinect Adventures games.	14 sessions. Two 60-min sessions per week for 7 weeks. Training program in small groups supervised by a physiotherapist.	Patients were assessed before and after the intervention. In addition, a follow-up assessment was performed at 4 weeks after treatment.	Balance	Mini-BESTest	Significant intragroup improvements in gait were found at the end of treatment in both the EG and CG (EG [*p* < 0.05] and CG [*p* < 0.005]), and these improvements were maintained over time.
				Gait	FGA	
				Cognitive Functions	MoCA	
**Yang, C.M. et al., 2020 [45]**	10 sessions. Two 45-min sessions per week for 5 weeks. Exercise with the Microsoft Kinect for Xbox 360 device (Microsoft, Washington, DC, USA), using “Your Shape: Fitness Evolved II” game software.	10 sessions. Two 45-min sessions per week for 5 weeks. Physical exercise program specifically designed to prevent falls in elderly women.	Patients were assessed before and after the intervention.	Balance	TUG	EG showed greater improvements compared to CG in dynamic balance (z = −2.307; *p* = 0.021). Both groups showed intragroup differences in the 30-s CST the FRT and OLS with OE. In addition, EG showed improvements one-leg standing test with CE (z = −2.803; *p* = 0.005) and the timed up and go test (z = −2.803; *p* = 0.005).
					FRT	
					One-leg stance test	
				Lower limb strength	30-s Chair Stand Test	

Abbreviations. ACT: Arm Curl Test; BBS: Berg Balance Test; CE: Closed Eyes; CG: Control Group; EG: Experimental Group; FRT: Functional Reach Test; MoCA: Montreal Cognitive Assessment; OE: Opened Eyes; QoL: Quality of Life; SF-36: 36 Items Short form Survey; TUG: Timed- UP and Go test.
